# A qualitative study of living with the burden from heart failure treatment: Exploring the patient capacity for self‐care

**DOI:** 10.1002/nop2.455

**Published:** 2020-02-08

**Authors:** Oda Karin Nordfonn, Ingvild Margreta Morken, Anne Marie Lunde Husebø

**Affiliations:** ^1^ Department of Public Health Faculty of Health Sciences University of Stavanger Stavanger Norway; ^2^ Department of Health and Caring Sciences Western Norway University of Applied Sciences Stord Norway; ^3^ Department of Cardiology Stavanger University Hospital Stavanger Norway; ^4^ Department of Gastroenterological Surgery Stavanger University Hospital Stavanger Norway

**Keywords:** capacity, heart failure, nurses, nursing, qualitative, quality of life, treatment burden

## Abstract

**Aim:**

To explore how patients with heart failure perceive their capacity to manage treatment and self‐care.

**Design:**

A qualitative descriptive study.

**Methods:**

Patients (*N* = 17) were recruited from a nurse‐led heart failure outpatient clinic from May–August 2017. Data were collected through individual semi‐structured interviews and analysed using systematic text condensation.

**Results:**

Three main themes were identified as follows: “Personal characteristics,” “Coping strategies” and “Emotional and informative support.” The first main theme contained the subthemes “inherent strength” and “maintenance of a positive attitude.” The second main theme included the subthemes “selective denial,” “ability to adapt by setting new goals” and “careful selection of information.” The third main theme contained the subthemes “support from health professionals enhancing patient capacity,” “support from next of kin in patients' self‐care” and “practical support and hope from peers.”

## 1 INTRODUCTION

Heart failure (HF) is a clinical syndrome causing a potential heavy burden from illness and symptoms (Ponikowski et al., 2016). HF patients are often enrolled in complex treatment regimens with a heavy medication load and demanding lifestyle changes (Kessing, Denollet, Widdershoven, & Kupper, 2016); additionally, treatment may become a burden (Eton et al., 2012; Gallacher, May, Montori, & Mair, 2011; May et al., 2014). This study is based on the burden of treatment (BoT) framework. BoT is defined as the “workload” delegated from health care to patients and the impact this “work” has on patients' well‐being and functioning (Eton et al., 2015; Gallacher, May, Langhorne, & Mair, 2018; May et al., 2014). Capacity is a central concept in the BoT framework (May et al., 2014). As patients balance the workload from treatment and self‐care with their capacity, their workload may exceed their capacity and create a BoT (May et al., 2014; Shippee, Shah, May, Mair, & Montori, 2012). Patients' ability to manage the BoT is important in affecting the outcomes and stability of HF disease (Eton et al., 2012; Shippee et al., 2012). However, capacity is an emerging concept and descriptions of capacity based on HF patients' experiences are sparse (Boehmer et al., 2016). Therefore, HF patients' experiences of capacity when facing treatment and self‐care is important. Moreover, we need to understand what HF patients consider to be sources of capacity and relief from the BoT.

## 2 BACKGROUND

Capacity is an evolving concept defined as the patients' abilities (e.g. personal, physical, mental, social, financial and environmental), resources and limitations that affect the patients' capability to address demands from health care and life with a chronic illness (Boehmer et al., 2016; Eton et al., 2012; Lippiett, Richardson, Myall, Cummings, & May, 2019; May et al., 2014; Shippee et al., 2012). Patient capacity is viewed as a complex and dynamic construct, as it includes both the individual patients' functional performance and depends on the social skills and social capital of patients and members of their social networks (Boehmer et al., 2016; May et al., 2014). Boehmer et al. (2016) described patient capacity as the achievement of the ability to reshape one's biography of life with chronic condition. In addition, patients' resources, their social networks, an environment of kindness, empathy, a feasible treatment plan and the ability to carry out actions required for health care and self‐care despite competing priorities affect capacity (Boehmer et al., 2016). Importantly, capacity is not a static entity but a dynamic accomplishment that is hindered or bolstered by psychological and social mechanisms and that varies depending on experiences of exacerbating events or stability (Boehmer et al., 2016; Lippiett et al., 2019). To the best of our knowledge, capacity is a concept that has not been previously described in HF patients; however, other notions such as capability for self‐care work as well as barriers and facilitators of self‐care have been used (Wanchai & Armer, 2018). Capability for HF self‐care relates to patient compliance and describes the level of adherence to treatment recommendations, acceptance of illness and occurrence of frailty. Low capability for HF self‐care is linked to reduced adherence to treatment and treatment recommendations (Mlynarska, Golba, & Mlynarski, 2018). Additionally, Wingham, Harding, Britten, and Dalal (2014) state that patients living with HF undergo transformations while adjusting to the life‐limiting, lifelong condition. This process is not a linear trajectory but a progression through different stages shaped by HF patients' attitudes, strategies and support, demanding constant effort to maintain health. Burdens related to treatment and self‐care in HF have been characterized in prior research as overwhelming, difficult work; and adherence to HF self‐care and treatment regimens has been reported to be low (Gallacher et al., 2011; Mlynarska et al., 2018; Riegel et al., 2019). Despite patients' knowledge of self‐care tasks, the burden of the illness and treatment generates problems in maintaining self‐efficacy, which is a personal attribute that changes over time by learning from attempts to manage the HF condition (Nordfonn, Morken, Bru, & Husebø, 2019; Riegel et al., 2019; Spaling, Currie, Strachan, Harkness, & Clark, 2015). None of the aforementioned notions fully captures the capacity term in the HF context. Therefore, how HF patients experience their capacity to manage treatment and what provides HF patients with relief from burden are important aspects to further address. The aim of this study was to explore how HF patients perceive the capacity to manage treatment and self‐care.

## 3 METHOD

This study had a descriptive, explorative qualitative design to understand HF patients' experiences of the capacity for management of treatment and self‐care using individual semi‐structured qualitative interviews.

### 3.1 Recruitment of participants

A purposive sampling strategy was used among the patient population of one nurse‐led HF outpatient clinic located in the western Norway (Polit & Beck, 2012). In the clinic, HF patients see their assigned HF nurses for a limited time for up‐titration of medical therapy at different time increments: every other week, once a month, every third month or once every 6 months. During the consultations, the nurses consult with a cardiologist for the adjustment of medications (McDonagh et al., 2011). A study nurse screened the patient list at the outpatient clinic including the eligible participants emphasizing the latest HF nurses' journal note in the medical record according to the New York Heart Association (NYHA) classification. The inclusion criteria were diagnosis of HF confirmed by echocardiography at least 3 months prior, NYHA class II or class III and age of 18–75 years. Patients were excluded if they were unable to speak Norwegian or suffered from cognitive impairment. Eligible participants were first invited by an invitation letter explaining the study and second, by an oral invitation from the HF nurse at the outpatient clinic. Patients who accepted the invitation were contacted by telephone to arrange a convenient date and time for the interview. Forty‐nine patients were eligible. Seventeen participants, 11 male and six female patients (Table 1: Participant characteristics), gave their written consent to participate in the study.

**Table 1:** Participant characteristics (*N* = 17)

Parameter	*N* (%)
Mean age, years (range)	62 (46–74)
Sex	
Male/Female (% male)	11/6 (65)
Marital status	
Living with spouse/partner	13 (77)
Living alone	4 (23)
Educational level	
Primary school	3 (18)
High school	11 (65)
College/university degree	3 (18)
Employment	
Working	7 (41)
Retired/disability pension	10 (59)
NYHA class	
II	11 (65)
III	6 (35)
Time since diagnosis, years (range)	2.5 (0.5–7)
Aetiology	
Myocardial infarction	3 (18)
Cardiomyopathy	7 (41)
Coronary artery disease	1 (6)
Hypertension	1 (6)
Tachycardia induced	1 (6)
Unspecified heart failure	2 (12)
Ventricular dysfunction	1 (6)
Mitral insufficiency	1 (6)
Comorbidities	
Asthma	1 (6)
COPD	2 (12)
Diabetes type 2	3 (18)
Kidney failure	2 (12)
Atrial fibrillation	5 (29)
Arthritis	1 (6)
Systemic sclerosis	1 (6)
Ventricular arrhythmias	2 (12)
None	4 (23)
Device	
ICD	8 (47)
CRT‐P	1 (6)
CRT‐D	4 (23)
None	4 (23)

Abbreviations: COPD, chronic obstructive pulmonary disease; CRT‐D, cardiac resynchronization defibrillator; CRT‐P, cardiac resynchronization therapy pacemaker; ICD, implantable cardioverter‐defibrillator; NYHA, New York Heart Association; Primary school, 9 years in Norway.

### 3.2 Data collection

Following the provision of informed written consent, each participant took part in a recorded semi‐structured interview in the hospital or a private setting between May–August 2017. The first author, a female nurse trained and with previous experience in the interviewing procedure, conducted all interviews. The interviews lasted for 30–90 min, with a mean time of 55 min. The interviews featured a series of open‐ended questions adapted from previous studies on capacity and burden of treatment (Eton et al., 2012; May et al., 2014; Sav, Salehi, Mair, & McMillan, 2017) (Table 2: Interview schedule). Demographic information, such as age, education level and marital status, time since diagnosis, NYHA class, device, aetiologies and comorbidities, was collected from the participants and from medical records after the interviews.

**Table 2:** Interview schedule

Theme	Main questions	Supporting questions
General introduction	Could you tell me about your health problems?	
Daily management of the disease	What do you have to do to take care of yourself and the disease? How do you cope in daily life with self‐management of the disease? When you think of all the things you have to do to take care of yourself, how will you describe that it affects you and your daily life? How much would you say handling the work you have to do in relation to you disease, takes in your life? Do you sometime feel that it is hard to do all these things to take care of your health? Do you ever skip any of the things you should do?	How do you monitor your symptoms? Do you weight yourself on a weekly basis? How do you manage the disease in daily life? What new skills have you learned to take care of yourself? Does it affect your work, social life, family life? Could you say something about how much time you spend on taking, organizing and collecting medication, organize and get to appointments and examinations? Could you say something about how much time you spend on exercise and diet, based on advice from health professionals? Do you perceive that you follow the advices you get?
Relationship with health professionals	Could you tell me about your relationship with different health professionals?	How is communication? Do you have any examples?
Social support	In relation to your heart failure, do you get any help from others? Does this ever cause any tension between yourself and others?	Who helps you? How do they help you? Could you tell me something about how you and your next of kin collect information, if there is somethings you wonder about in relation to your disease?
Emotions	For some, the personal work they have to do in relation to the disease is emotionally challenging. Is this something you can relate to? Is there something you do to keep you spirits up?	
Burden of Treatment	*Explanation of burden of treatment.* How do you experience self‐management of your disease, thinking about this burden of treatment?	Can you in some way relate to it? Are there other things that makes it difficult to live with the disease? Do you experience a continuity of care in relation to your disease?
Capacity	If you picture a weight scale, on the one side, you have your disease and on the other side, you have your capacity to handle the disease, what do you think about the relationship between them?	How do you perceive your capacity in daily life?
Ending	Is there something important you want to add?	

### 3.3 Rigour

To obtain rigour during the data collection process, the same interviewer conducted all of the interviews, ensuring authenticity through prolonged engagement and continued until no new elements were obtained from the analysis of the interviews and an agreement was made among the authors that saturation was reached (Malterud, Siersma, & Guassora, 2016; Polit & Beck, 2012). The interviewer was not clinically involved in the participants' care.

Coding, categorization and generation of themes were managed by the three authors to ensure that the process was rigorous and that the themes were consistent with the data. All authors independently analysed the transcripts and then discussed their findings with the research group until an agreement was reached on the final analysis. Peer checking was used to supplement the interviewees' proofreading efforts to ensure reflexivity, a hallmark of qualitative research and to increase reliability (Lincoln & Guba, 1985; Rolfe, 2006). The researcher kept an audit trail during the study to keep careful documentation of the work in the form of a journal with interview notes and observation notes. This was done to make thought process explicit, identify and reflect on personal knowledge and experience and consider how these processes may have had an effect on the research and analytical process (Carlson, 2010; Sutton & Austin, 2015).

Clear, precise descriptions of the context, the selection and characteristics of the participants, the collection of data and the process of analysis are provided in this paper and extensive participant quotes are included in the findings section to ensure credibility (Polit & Beck, 2012). The Consolidated Criteria for Reporting Qualitative Research (COREQ) 32‐item checklist (Tong, Sainsbury, & Craig, 2007) was used as a guide to achieve comprehensive and explicit reporting of the study (see File S1).

### 3.4 Analysis

The first author transcribed the audio recordings verbatim. After being checked for accuracy by comparing the transcripts to the audio recordings, the transcripts were stored in the computer software program NVivo 12 (QSR International Pty. Ltd.). NVivo 12 was used to aid data management and to enable a systematic approach to analysis. Data analysis was carried out by the use of systematic text condensation (STC) (Malterud, 2012, 2017). STC consists of 4 phases of analysis. In phase one, the primary goal was to obtain an overview and create preliminary themes. In phase two, the themes and subthemes were generated based on preliminary themes. In phase three, the participants' quotes, as meaning units, were organized in a hierarchical theme structure and condensed. Phase four involved synthesizing the most nuanced themes identified in phases two and three, resulting in the three main themes (see Table 3: Themes and subthemes). A translator proficient in both languages translated the participants' quotes from Norwegian to English to preserve the patients' voices and the meaning of the content.

**Table 3:** Themes and subthemes

Meaning units (a sample)	Subthemes	Themes
I think I was born with a will to fight. If anything else had happened, I probably would have managed that as well. I think I have the energy for that too. I have this (will to fight) in me	Inherent strength	Capacity through personal characteristics
I've never seen the advantage of sitting down and feeling sorry for myself. I mean, this illness is also a part of how life has become, with heart failure. I cannot sit down, feeling sorry for myself. That would only make me sicker. One has to live life as best as one can and I want to live	Maintenance of a positive attitude about life	
I do not talk about it (heart failure).Okay, I know I have heart failure, but I don't want to focus on that. Instead, I focus on other things. That's how it is	Selective denial	Capacity through coping strategies
Other people have hobbies, and this (heart failure) is mine. Now it's up to me to exercise more. We have a mountain cabin, which takes a one and a half hour walk, and I haven't been there these last 2 years. My goal is to be able to walk that distance	Ability to adapt by setting new goals	
I do not want to be a user of medical information found on the internet because I am not able to judge on the information I find there. I know the internet and the problem with all the information online. To me it is a dangerous field to navigate. I have told my GP that I trust him and other experts on the field	Careful selection of information	
The follow‐up visits at the outpatient clinic makes me feel safe. I attend the clinic every other or every third months and that's fine. I feel so well looked after and that's why I feel so safe	Support from health professionals enhancing patient capacity	Capacity through emotional and informative support
I often forget many things so my wife accompanies me to my appointments. She tends to remember things I forget. My wife's support means a lot	Support from next of kin in patients' self‐care	
I met another man and his wife at a (heart failure) course where you can bring your spouses. He talked about having the exact same problems that I had and said it was the medication causing my problems	Practical support and hope from peers	

### 3.5 Ethics

Ethical permission was obtained from the Regional Committee for Medical and Health Research Ethics (REK no. 2017/75). Verbal and written information regarding the purpose of the interview study, the procedure and the right to decline or withdraw at any time was provided to all potential participants. Information about the study was repeated to participants after inclusion. All participants received information to contact the ward staff at the outpatient clinic if the interview caused the need for further conversation. No identifying patient information was stored together with the data. Anonymity and confidentiality were secured by labelling interview transcripts by interview number and participant identification key, and data were stored separately on the hospital server.

## 4 FINDINGS

A total of 17 patients were included in this study. The study participants ranged in age from 46–74 years (mean 62 years); 11 were male and six were female. Thirteen of the participants lived with a spouse or partner; the other four lived alone.

### 4.1 Theme 1: Capacity through personal characteristics

This theme highlights how patients cultivated a set of personal characteristics linked to the capacity to deal with HF and self‐care. Personal characteristics included the subthemes “inherent strength” and “maintenance of a positive attitude about life.”

#### 4.1.1 Subtheme 1.1: Inherent strength

Most patients expressed living with HF as a heavy burden in their lives and that their inherent personal strength was one of the main reasons for their current capacity to manage the burdens from a severe HF diagnosis and self‐care. The patients described this strength as an attribute they had developed throughout their entire lives and not a trait developed during the time of the HF diagnosis:

I think I was just born like this. I will not let this disease destroy me. I cannot sit down and feel sorry for myself. Suddenly, your whole life has passed and you have just sat there. (P 11, NYHA II)

In addition, the patients' personal strength gave them the capacity to endure the workload of treatment and self‐care combined with both work and leisure time. Some of the participants expressed periods of sadness and depression related to having HF, but these episodes were explained as temporary and as short occurrences of bad moods that their personal strength gave them a capacity to overcome to maintain their well‐being:

I have always thought that if you have to do something, you just have to do it. I think in some way I have a very strong spirit, so I won't let the disease affect me in that way, getting miserable. You just have to roll up your sleeves and keep up the fight. Otherwise, you end up depressed. I think I have a special strength to do that, not letting negative energy affect me. (P 6, NYHA II)

#### 4.1.2 Subtheme 1.2: Maintenance of a positive attitude about life

Patients described gaining capacity through being able to maintain a positive attitude despite their severe HF diagnoses. Several participants expressed that HF is a challenging disease; however, all of the participants highlighted that whining and feeling sorry for themselves drained them of energy and was something that they tried to avoid. The patients saw complaining as an unnecessary use of energy and gained increased capacity by focusing on how to persist and improve their health:

I mean, I can't spend my time thinking, “Oh no, now I have to take my medicine. Oh no, now I have to go to the pharmacy. Oh no, now I have to take blood samples.” There is no use in thinking like that. That's just a part of it [the HF]. There's no use in thinking of it like that. One has to think that, “I can get to the pharmacy and get the medicine. I can get those blood samples, too”. That's my job, a part of my life. (P 17, NYHA III)

The participants stated that they had gained capacity by taking 1 day at a time, enjoying life and focusing on the bright side of their small and large accomplishments:

Regardless of everything, I feel that I am lucky because I can manage everything. When I think of the whole situation, I have actually managed it. I am so grateful for that. I think of it every night, how lucky I am. My children and grandchildren are healthy. Therefore, I'm thankful for a lot of things every single day. (P 14, NYHA III)

### 4.2 Theme 2: Capacity through coping strategies

This theme incorporates the subthemes “selective denial,” “ability to adapt by setting new goals” and “careful selection of information.” The theme highlights the capacity‐giving coping strategies the participants used in their everyday lives dealing with the burden of illness and self‐care.

#### 4.2.1 Subtheme 2.1: Selective denial

Many of the participants described their coping as involving some degree of denial due to the severe illness. Several of the patients reported having chosen to ignore their illness and their health issues for some time as a way to survive and to manage in everyday life:

I didn't want to know about how bad my heart was. Because you just want to put your head in the sand and say, “No, this is not me; I don't have such severe heart failure.” You don't want to hear it. (P5, NYHA III)

By overlooking the disease and telling themselves that they did not have such a severe illness, they gained capacity to cope with HF. Some participants expressed this increase in capacity as something they did unconsciously to preserve themselves from the severity of the disease and to keep up their good spirits to maintain an ordinary life, while some expressed it as a conscious action:

I probably repress it [thoughts about the disease] sometimes. I think you live in some sort of a denial and you don't want to understand more than you think is good. Maybe forget and deny it? One can't go around and think of this all the time. It is better to live a normal life. (P 6, NYHA II)

#### 4.2.2 Subtheme 2.2: Ability to adapt by setting new goals

The HF patients spoke of adapting to their new life situations as a very important step in gaining capacity to manage the disease, treatment responsibilities and self‐care. The patients expressed the importance of accepting, reviewing and reconsidering their lives. Several patients related coming to a new understanding of how life was going to be, taking things more slowly and allowing themselves to rest during the day. Moreover, many of the participants highlighted keeping themselves busy during the day by setting small goals and attainable tasks for themselves as important for capacity. The ability to establish new achievement goals as a person with HF diagnosis was expressed by the participants as another strategy for gaining the capacity to manage HF:

I don't push myself any longer when I'm outdoor walking. I really don't. Instead, I take things more slowly now. Sometimes I think I take things too slowly and do not go for a walk at all. (P 10, NYHA II)

The patients expressed that their new life goals had to be attainable, such as being able to walk a certain distance or spend time at a mountain cabin. Others expressed a desire to be able to perform their old sports again or climb a mountain. One patient talked about how he now reconsidered how he used his energy to be able to spend more time with grandchildren:

You set goals for yourself, like being able to spend more time with your grandchildren; to enjoy yourself. To be able to reach those goals, you have to do a job. Like taking it more slowly, not work so hard. (P 16, NYHA III)

#### 4.2.3 Subtheme 2.3: Careful selection of information

All of the participants expressed the importance of being informed about current and future plans for treatment in building capacity to manage their conditions. Several patients explained that only a limited amount information was required, especially the information provided health professionals, to enable them to keep up with managing the disease. Several patients avoided the Internet as a source of information, explaining that too much information scared them:

I want to know as little as possible. I'm a bit afraid. I'm not afraid of dying, no such thing. I'm afraid of getting more nervous than I already am. Because I feel I have enough as it is. If I will get a lot of information, I will go completely mad. That's what I'm most frightened of. How I will react. That's why I want to know as little as needed. (P 13, NYHA III)

### 4.3 Theme 3: Capacity through emotional and informative support

The final theme was associated with the patients' experiences of emotional and practical support as a strong relief from the burden derived from treatment and self‐care and a manner of regaining capacity. The support they gained from health professionals, next of kin and peers was important in their everyday capability for HF self‐care.

#### 4.3.1 Subtheme 3.1 Support from health professionals enhancing patient capacity

Most participants described distress related to HF treatment and self‐care. They also noted their valued relationships with the HF outpatient clinic and specialist HF nurses as a source of capacity. The participants spoke of their connection with the outpatient clinic that it made them feel safe and under surveillance. This surveillance was described as a way to ensure sustained wellness, gain capacity and obtain the best treatment to maintain the best possible health. The patients expressed their attendance at the outpatient clinic as having somebody else to share the responsibility of a severe illness with and having help watching for signs of deterioration:

In my opinion, I think they [the outpatient clinic] are good at separating the important things from the not so important things in what I say. They are good at sorting out all the things I tell them, what is relevant and what is not. I leave it up to them to sort out and interpret the signals and to alarm if it's needed. That's my safety. Instead of being prepared for everything, I leave it up to these appointments. That's my way of handling things. It's more likely that the nurse can separate things, see signals of worsening and make sufficient medication adjustments. I think of it as checkpoints and that I can relax between those checkpoints. (P 4, NYHA II)

Although the outpatient clinic was supposed to be a limited affiliation with the healthcare system, the patients expressed the desire to stay in contact with the clinic as long as possible:

The heart failure outpatient clinic has been a good place to be. It's very safe to get a check‐up by the nurse. I don't want to quit going there. She [the nurse] said it could be a while between the appointments, but I've said that no matter what, I would like to keep going there. I've really received help going there. (P 13, NYHA III)

#### 4.3.2 Subtheme 3.2 Support from next of kin in patients' self‐care

Most patients expressed support from spouses, children, grandchildren and friends as crucial to maintaining capacity and enduring the burdens of treatment and self‐care. Next of kin were seen as a counterbalance, representing what the patients associated with normal life. Patients' spouses were seen as solid supporters in everyday life and as companions carrying some of the burden of severe illness with them. Children and grandchildren helped both in the practical tasks of self‐care, such as remembering to take prescribed medicating and to go to appointments and helping patients maintain a good diet and exercise. In addition, patients described the family as providing emotional support by calming them when they were afraid and supporting them during medical appointments:

I am lucky to have such a wife. If I hadn't had her, I probably wouldn't be sitting here now. So, thank goodness, I'm lucky to have her. Nobody knows me as well as my wife and if something happens, she calms me down and I feel better. Thank goodness, I have her. (P 3, NYHA II)

#### 4.3.3 Subtheme 3.3 Practical support and hope from peers

Several participants considered other HF patients a source of capacity because they had given them information and practical knowledge they could not gain from others. Several patients related that learning about peers' personal experiences with HF was useful, inspirational and comforting, giving hope for the future:

I met a young girl, 8 years younger than me. She talked about experiencing the same as me 7 years ago. “Seven years ago,” I thought. That was the first time I started to think “Can I live for 7 years?” That gave me hope and made me realize I wouldn't die tomorrow. (P 4, NYHA II)

## 5 DISCUSSION

This study describes aspects of how HF patients perceive the capacity to manage the burden arising from treatment and self‐care. The analysis reveals that the patients view their inherent strength and the use of coping strategies as well as social support as their main sources of the capability to manage the burdens of treatment and self‐care.

### 5.1 Capacity and personal characteristics

The participants in this study expressed their personal characteristics, hence their inherent strength and their ability to keep a positive spirit, as an important source of the capacity to manage the burdens of treatment and self‐care. These findings align with Lippiett et al. (2019), who stated that capacity constitutes the emotional and personal resources that affect patients' abilities to carry out the work of self‐care at a personal level. HF patients experience stressors related to physical capabilities and their sense of self (Harkness, Spaling, Currie, Strachan, & Clark, 2015). In response to these stressors, Harkness et al. (2015) found that personal resources such as attitudes and personal characteristics influenced patients' self‐care abilities and capacity. Falk, Wahn, and Lidell (2007) and Ridgeway et al. (2014) similarly argued that HF patients' capacity for a demanding life situation depends on attitudes about life and personal characteristics enabling them to manage their situations. However, as patient capacity is not just a mobilization of available resources (Ridgeway et al., 2014), we need further knowledge about how to increase and cultivate individual capacity.

### 5.2 Capacity and coping strategies

The findings in our study point towards HF patients alternating between different coping strategies. The participants described using both denial and repression of the disease and setting new self‐care goals. Alternating between contradictory coping strategies might seem conflicting. However, patients' uses of what sometimes may be viewed as negative coping strategies might be a way of surviving difficult phases. Harkness et al. (2015) reported similar findings in terms of what they called a perception‐based strategy, a cognitive, emotional and psychosocial response to adjusting to living with a chronic condition. By the use of denial or ignoring of symptoms, a rejection of self‐care was also a part of gradually redefining life with HF. Moreover, our findings relate to what Wingham et al. (2014) called “selective denial,” when patients recognize HF as a serious illness but deny its personal significance. As a result, patients fail to notice the consequence of their non‐engagement in HF self‐care. A low acceptance level of the HF diagnosis, which is seen in denial, is related to a low capability for self‐care (Mlynarska et al., 2018). Therefore, such coping strategies may hinder patients' abilities to practice proper self‐care. However, despite periods of selective denial, Wingham et al. (2014) found that HF patients experience a transformation process as an adjustment to living with a chronic condition. This process is a non‐linear path through the stages of disruption, sense making, reaction, response and assimilation to become a person living with HF. Trusted HF nurses focusing on transformation processes in the HF trajectory may help patients in developing coping strategies, increasing capacity and acceptance and setting new goals for their lives with the HF diagnosis.

In the present study, careful selection of information also represented a way of coping. Although medical information is easily accessible through the internet, several of the participants noted restricting which information they would trust. Finding, understanding and trusting medical information is identified as a treatment burden in the long‐term illness context (Eton et al., 2017). The Internet has changed the health information landscape and an increasing number of people seek medical information online (Jacobs, Amuta, & Jeon, 2017). Linn et al. (2019) studied information‐seeking behaviour in chronically ill patients and found that compared with patients who did not use the internet to prepare themselves before the consultation, those who used the Internet before a consultation with a nurse specialist were more likely to have more negative beliefs about medication and poorer medication adherence. By initiating dialogue with HF patients on their information‐seeking behaviour and guiding them towards safe information sites online, HF nurses may affect patients' capacity to better cope with a burdensome and lifelong disease.

### 5.3 Capacity and social support

The patients in our study described support from health professionals, next of kin and peers as important to their capacity to manage the burdens of treatment and self‐care. Our findings indicate that HF nurses at outpatient clinics are a major contributor to patients' feelings of safety and security during an ongoing, chronic condition. Patients described the outpatient clinic as a surveillance programme and checkpoint that provided comfort and security took the burden off their shoulders and made them worry less in between appointments. As HF does not involve an endpoint in the treatment regimen but rather is a condition that has to be managed over a long period of time (May et al., 2014), the therapeutic relationship with the HF nurse is highlighted in our study as relieving patients from their self‐care workloads. Guidelines (Ponikowski et al., 2016) recommend that patients attend outpatient clinics as a way of up‐titrating medication to optimal medication levels for a limited time. Our findings may indicate that patients view the outpatient clinic as providing something more than just medication titration. The feeling of being in safe hands at an outpatient clinic may help and guide patients in the process of transformation to maintain an acceptable state of health (Dubouloz et al., 2010; Spaling et al., 2015; Wingham et al., 2014). It is clear from our findings that HF nurses play an important role in motivating HF patients to engage in self‐care, respond effectively to treatment demands and boost their capacity. Wanchai and Armer (2018) recommend several methods HF nurses can use to increase patient capacity for self‐care as part of a supportive‐educative system comprising support, guidance and teaching. In the present study, the HF patients perceived the HF nurse as strengthening their abilities to monitor their health conditions, which is important to their feelings of security and self‐efficacy. More research on how the relationships between nurses and HF patients shape capacity for self‐care is required.

In addition to the HF nurse and the outpatient clinic, the findings also indicate the HF patients' next of kin and peers as important contributors to capacity for self‐care. These findings align with prior research on the importance of close relationships in long‐term illness and highlight the need for health professionals to be aware of the importance of care from next of kin (Kang, Li, & Nolan, 2011; Strøm, Andersen, Korneliussen, & Fagermoen, 2015). Peers were reported as a source of hope, practical advice and normalization of the condition. Lockhart, Foreman, Mase, and Heisler (2014) found that HF patients took better care of themselves and were motivated by engaging with peers. As social support may be an independent factor in mortality and morbidity in HF, talking with, sharing and learning from other HF patients is important (MacMahon & Lip, 2002). Therefore, HF nurses should engage HF patients in peer partnerships as a tool for increased self‐care and capacity.

### 5.4 Implications for practice

Our findings suggest that healthcare professionals should focus on and pay attention to the patients' ability to manage their illness, from treatment and self‐care. Initiating a dialogue with the patients, focusing on both the patients' resources and limitations of their social network capacity, might help patients go through the transformation process and achieve a normalization of the chronic illness. Engaging and helping patients manage the changing dynamic in their capacity for self‐care work, acknowledging the natural fluctuations of energy that follows a severe diagnosis and the need for relief and providing help and support from healthcare professionals and others are crucial aspects of health management.

### 5.5 Limitations

This study has some methodological limitations. First, credibility may have been affected, as we did not allow the recipients to check our interpretation of the data. To secure trustworthiness and validity, we used peer checking to avoid bias in the study. However, participants' proofreading of transcripts could have contributed further to the rigour of the data analysis. Second, this study was conducted only in one outpatient clinic in Norway. This may have resulted in the sampling of both the least burdened and the best cared for patients. Therefore, patients not attending outpatient clinics might describe their capacity differently. Out of the 49 eligible patients, only 17 agreed to participate. Patients who declined to participate indicated that they felt overburdened by their disease and had too much on their plate. Consequently, other significant findings on capacity may have been prevalent in HF patients not attending outpatient clinics and patients with NYHA class IV.

In addition, 11 of the 17 participants were male, which may have caused an unintended gender bias (Affleck, Glass, & Macdonald, 2013). Still, as men are reported to have a higher incidence of HF across all ages (Rosengren & Hauptman, 2008), our study may contribute with important knowledge on male HF patients' experience of capacity. However, to account for gender bias in research, we could have recruited a strategic sample of participants to secure equal representation in the study population.

## 6 CONCLUSION

In conclusion, this study identified elements that HF patients described as enhancing their capacity for treatment and self‐care through a range of factors on personal, coping and support levels. Through different coping strategies, such as selective denial and setting new goals and through what the participants perceived as inherent strengths and personal characteristics, the HF patients both attained and gained capacity. In addition, our findings demonstrate the importance of the perception of being in safe hands through the support of trusted health professionals, the care of next of kin and hope provided by peers. Patients and their social network must navigate and coordinate their different and sometimes complex treatment regimens, which, with low capacity, may lead to disruption and poor clinical outcome (Demain et al., 2015; Eton et al., 2012; May et al., 2014; Shippee et al., 2012). By investing in improving HF patients' capacity and helping manage their workloads from treatment, HF nurses may promote better experiences of illness, more effective healthcare consumptions and better healthcare outcomes (May et al., 2014). HF nurses should be more aware of their role in HF patients' process of transformation and in the dynamic work of building the capacity for treatment and self‐care.

## CONFLICT OF INTEREST

The authors report no conflict of interest.

## AUTHOR CONTRIBUTION

All authors have contributed significantly and are in agreement of the content of the manuscript.
